# Physiology and metabonomics reveal differences in drought resistance among soybean varieties

**DOI:** 10.1186/s40529-022-00339-8

**Published:** 2022-03-25

**Authors:** Xiyue Wang, Yongping Li, Xiaojing Wang, Xiaomei Li, Shoukun Dong

**Affiliations:** 1 College of Agriculture, Northeast Agricultural University, Harbin, 150030 China; 2 Harbin Academy of Agricultural Science, Harbin, 150029 China

**Keywords:** Soybeans, Drought stress, Metabolomics, Physiological indicators

## Abstract

**Background:**

The soybean is an important food crop worldwide. Drought during the first pod stage significantly affects soybean yield, and understanding the metabolomic and physiological changes in soybeans under drought stress is crucial. This study identified the differential metabolites in initial pod stage soybean leaves under polyethylene glycol-simulated drought stress, using ultra performance liquid chromatography and tandem mass spectrometry, and the physiological indexes related to drought resistance.

**Results:**

Physiologically, drought resistance also generates enzyme and antioxidant activity; levels of superoxide dismutase, peroxidase, and catalase first increased and subsequently decreased, while those of soluble sugar, soluble protein, malondialdehyde, and proline content increased in both varieties. The contents of CAT, proline and soluble sugar in Heinong 44 (HN44) were higher than those in Heinong 65 (HN65), and the contents of MDA were lower than those in HN65. In metabolomics, the OPLS-DA model was used to screen different metabolites. KEGG analysis showed that the two varieties resisted drought through different ways. Amino acid metabolism and lipid metabolism play a key role in drought resistance of the two varieties, respectively. TCA cycle was one of the core pathways of drought resistance in two varieties. Changes in the content of l-Asparagine and citric acid may be one of the reasons for the difference in drought resistance between the two varieties.

**Conclusions:**

We think that the reasons of drought resistance among soybean varieties are as follows: the main metabolic pathways are different under drought stress; the contents of metabolites in these metabolic pathways are different; some physiological indexes are different, such as MDA, CAT, proline content and so on. Our study enhances the understanding of the metabolomic soybean drought stress response and provides a reference for soybean drought resistance breeding.

**Supplementary Information:**

The online version contains supplementary material available at 10.1186/s40529-022-00339-8.

## Introduction

The soybean [*Glycine max* (Linn.) Merr.] was domesticated in China 3000–5000 years ago and is one of the most widely cultivated crops in the world (Prince et al. 2020; Zhao et al. 2021b). The soybean is also an economically important legume crop that is cultivated worldwide to obtain vegetable oil and as a mineral and protein resource (Khan et al. 2021; Otie et al. 2021; Anderson et al. 2019). Due to its nutritional components and pharmacological value, it is also processed into traditional Chinese health foods (Wang and Komatsu 2018). Soybean production has increased in the last 4 decades (Müller et al. 2021). In 2018, approximately 80% of the world’s harvested soybeans was produced in the United States (35%), Brazil (34%), and Argentina (11%), a total equivalent to nearly 350 million tons, and production continues to show steady growth (FAO 2018; Łangowski et al. 2021). However, soybean production will need to increase by 70 percent over the next few decades to meet the growing human demand for plant protein (Godfray et al. 2010; Tilman et al. 2011). Despite steady soybean yield increases due to genetic and management improvements, yields have been negatively impacted by various environmental pressures such as salinity and temperature fluctuations, droughts, flooding, metal toxicity, pollutants, and ultraviolet radiation. These stressors adversely affect plant performance by disrupting cell function and metabolic activity. However, some studies have demonstrated that the effects of environmental stress, often characterized by complex factors, depend on the type and level of stress and the plant genotype (Hasanuzzaman et al. 2016).

Plant life cycles and reproduction are greatly affected by adverse environmental conditions, particularly drought, one of the most common environmental factors that affect productivity (Žádníková et al. 2016; Thayale Purayil et al. 2020). In many parts of the world, drought incidence and severity are expected to increase, placing considerable pressure on global agricultural production (Zia et al. 2021). In recent decades, many regions of the world, including most parts of Asia, have experienced major drought events, which are expected to intensify and present extreme agricultural challenges in such areas (Lobell et al. 2011). Drought stress decreases maize and wheat yields by 40% and 21%, respectively, and significantly reduces drought-resistant wheat yield (Daryanto et al. 2016; Anwaar et al. 2020). Drought and other abiotic stresses have impacted agriculture-based income, while urgently needed sustainable food production remains unrealized (Qaseem and Wu 2020; Hrmova and Gilliham 2018; Hrmova et al. 2020; Baillo et al. 2019). Drought stress is a serious nonbiological factor that can quickly and substantially alter the plant morphology, physiological, and molecular processes (Nabi et al. 2021). Sheteiwy et al. (2021) demonstrated that the contents of soluble sugars, lipids, proteins, and oils in soybean decreased under drought stress. Therefore, studying the response of plants to drought from the aspects of plant morphological structure, physiology and biochemistry, and metabolites can deeply understand the mechanism of plant drought tolerance, which is also of great significance for breeding new drought-tolerant varieties.

Numerous analytical platforms such as nuclear magnetic resonance spectroscopy, capillary electrophoresis-mass spectrometry, gas chromatography-mass spectrometry, and liquid chromatography-mass spectrometry (LC–MS), are required to study plant metabolites due to their wide range of chemical compositions and concentration (Selegato et al. 2019; Chen et al. 2011; Wang et al. 2015; Dai et al. 2019; Li et al. 2019; Ghosson et al. 2018). LC–MS is currently the most popular platform for plant metabolomics due to its speed, sensitivity, wide molecular weight window, and capping range, which allows for the analysis of numerous plant metabolites with concentrations differing by several orders of magnitude (Zheng et al. 2021). Plant metabolite variety and quantity can reflect environmental adaptability (Töpfer et al. 2015). Jia et al. (2020) reported that carbohydrate, amino acid, lipid, and energy metabolism are involved in a drought response common to two poplar trees. Citric acid circulation is significantly inhibited to save energy, and a variety of carbohydrates such as osmotic agents and protectors are induced to mitigate the adverse effects of drought stress. Kang et al. (2019) revealed that tryptophan, valine, citric acid, fumaric acid, and malic acid accumulate more in leaves under drought stress. Zhao et al. (2021a) demonstrated the plant metabolomic changes exhibited under PEG-simulated drought stress, including those involving glycolysis, phenolic metabolism, tricarboxylic acid cycle, glutamate-mediated proline biosynthesis, urea cycle, amino acid metabolism, unsaturated fatty acid biosynthesis, and the met salvage pathway. A study of the sesame plant under drought stress exhibited high accumulation of metabolites including the plant hormone abscissic acid, 4-aminobutyric acid, organic acids such as glutaraldehyde and 2-methyl citrate, and amino acids such as tryptophan, phenylalanine, valine, leucine and tyrosine. In contrast, drought stress decreased levels of nucleosides and nucleotides, including guanosine, and uridine (You et al. 2019). Moradi et al. (2017) identified proline, betaine, mannitol, sorbitol, ascorbic acid, jasmonic acid, unsaturated fatty acids, and tocopherol as key drought-resistant metabolites in thyme research.

Varieties with strong resistance are often favored in the breeding process, so it is very important to explore the differences in drought resistance among different varieties. In this study, polyethylene glycol (PEG-6000) was used to simulate drought conditions in treated soybean leaves at the initial pod stage. The changes in metabolites were comprehensively analyzed by ultra-performance liquid chromatography and tandem mass spectrometry (UPLC-MS/MS). Our results enhance the understanding of the soybean plant’s metabolic response to drought stress and lay the foundation for breeding drought-resistant soybean varieties.

## Materials and methods

### Experimental materials and treatment

#### Sand culture

The soybean plants were potted in a sand culture, each made from a gray cylindrical barrel with a diameter of 20 cm and a height of 40 cm, the bottom of which was removed and covered with gauze, and sand washed with water was placed into each barrel. Six similarly sized, disease- and insect pest-free seeds of each of the selected drought-resistant soybean variety Heinong 44 (HN44) and the drought-sensitive soybean variety Heinong 65 (HN65) were sown in the sand culture, one variety per pot. Seedlings were separated into three plants per pot, repeat three times, with one pot of three used as a control, and the other for drought stress treatment. Plants were irrigated with 500 mL of water daily until true leaves appeared, then with 500 mL of Hoagland nutrient solution. The leaves were completely expanded to the initial pod (R3) stage, and 500 mL of Hoagland nutrient solution was irrigated once per day.

#### Drought stress treatment in R3

To simulate drought stress, the three soybean plants in each drought treatment (DS) group were treated in the morning and evening with 500 mL of a mixture containing 15% PEG-6000 dissolved in the Hoagland nutrient solution. The three soybean plants of each control group (CK) were watered as normal, without added stress treatment. So the experiment consisted of four groups in total: HN44-CK, HN44-DS, HN65-CK, HN65-DS. Leaf sampling was initiated between 8:00 and 9:00 a.m. on the fourth day of stress treatment, with the removal of the top 2 or 3 leaves. Each of the three plants within each CK and DS group were similarly harvested, and leaves were stored at – 80 °C for later testing. Sampling was repeated each day through day 10. Each treatment has three replicates.

### Determination of physiological indicators

From each sample, 0.5 g of leaf material was cut and placed into a pre-cooling bowl, with a small amount of quartz sand and 10 ml of pre-cooled phosphate buffer (pH = 7.8, 0.05 mmol/L) and ground in an ice bath. Subsequently, 10 mL of the pulp was centrifuged at 10,000 rpm for 20 min at 25 °C and stored at 0–4 °C. Activity levels of superoxide dismutase (SOD), peroxidase (POD), and catalase (CAT) were determined by nitroblue tetrazolium assay, guaiacol assay, and UV–visible spectrophotometry, respectively. The contents of MDA and soluble sugar were determined by thiobarbituric acid assay, while the contents of soluble protein and proline were determined by Coomassie brilliant blue G-250 staining and ninhydrin colorimetry, respectively. To ensure the accuracy of the data, each value is the average of three measurements. These methods were performed in accordance with the experimental method of Dong et al. (2019).

### Experimental process of metabolomics

Sample preparation, extract analysis, metabolite identification and quantification were carried out in Wuhan metware Biotechnology Co., Ltd. (www.metware.cn) according to standard procedures, which were previously described in detail by Yuan et al. (2018), Zhang et al. (2019a) and Zhang et al. (2019b). To search for differential metabolites, using an orthogonal partial least squares discriminant analysis (OPLS-DA), which combines orthogonal signal correction and partial least squares discriminant analysis, and decomposes the X matrix information into two types: y-related and independent, was used to extract the components of the independent variable X and dependent variable Y, calculate the correlation between components, and filter the differential variables by removing irrelevant differences (Bylesjö et al. 2006). After log2 transformation of the original data, OPLS-DA was processed by mean centering using the MetaboAnalystR package OPLSR. Anal function in R software. Analysis of metabolic data based on OPLS-DA model, drawing score maps of each group, further showing the differences between each group (Thévenot et al. 2015).

### Relevant analysis software

The data were drawn using Microsoft Excel 2013, PCA coefficient using R (base package) 3.5.0, heat map using pheatmap (R) 1.0.12, and OPLS-DA using MetaboAnalyst R (R) 1.0.1.

## Results

### Physiological changes of two soybean cultivars under drought stress

**Fig. 1:**
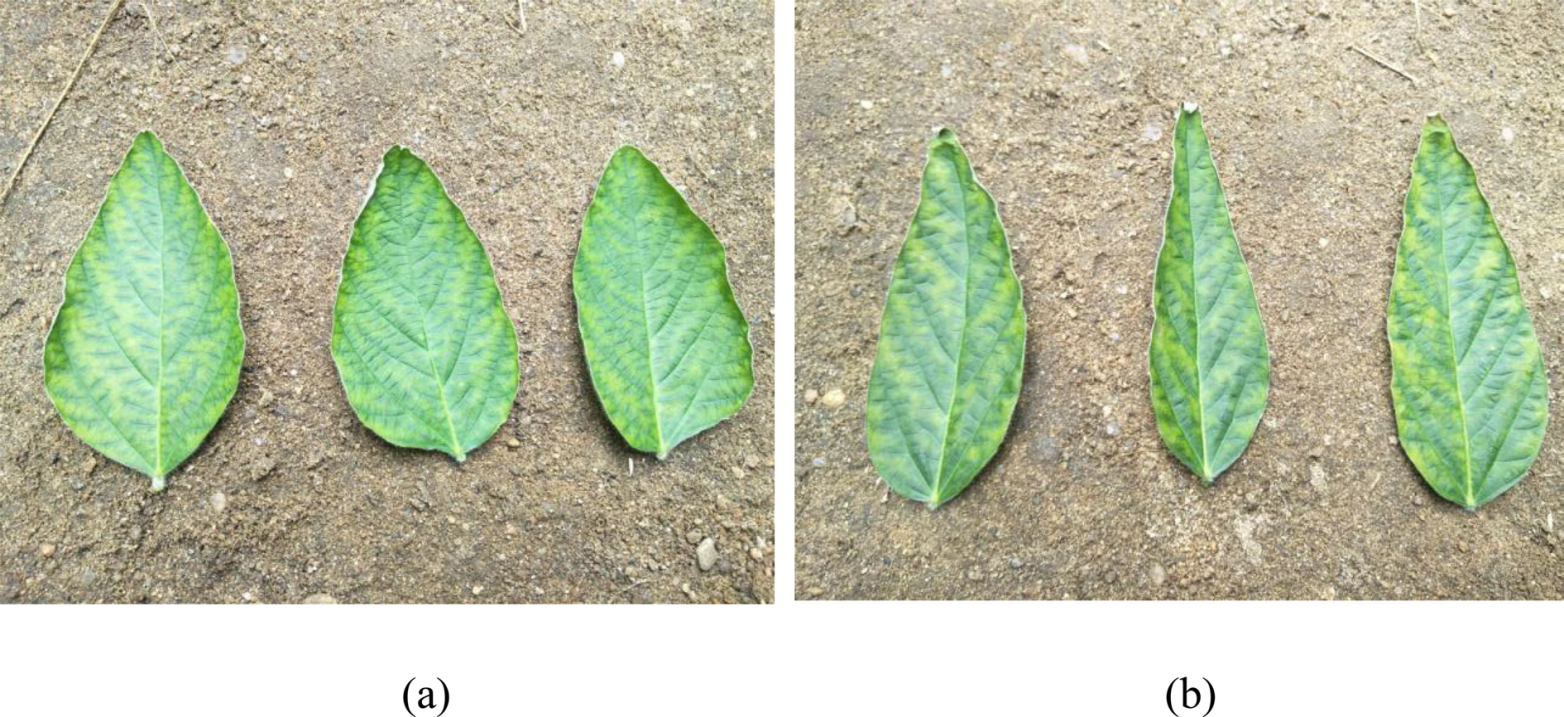
Leaf status of two soybean varieties under drought stress. **a** drought-resistant HN44; **b** drought-sensitive HN65

**Fig. 2:**
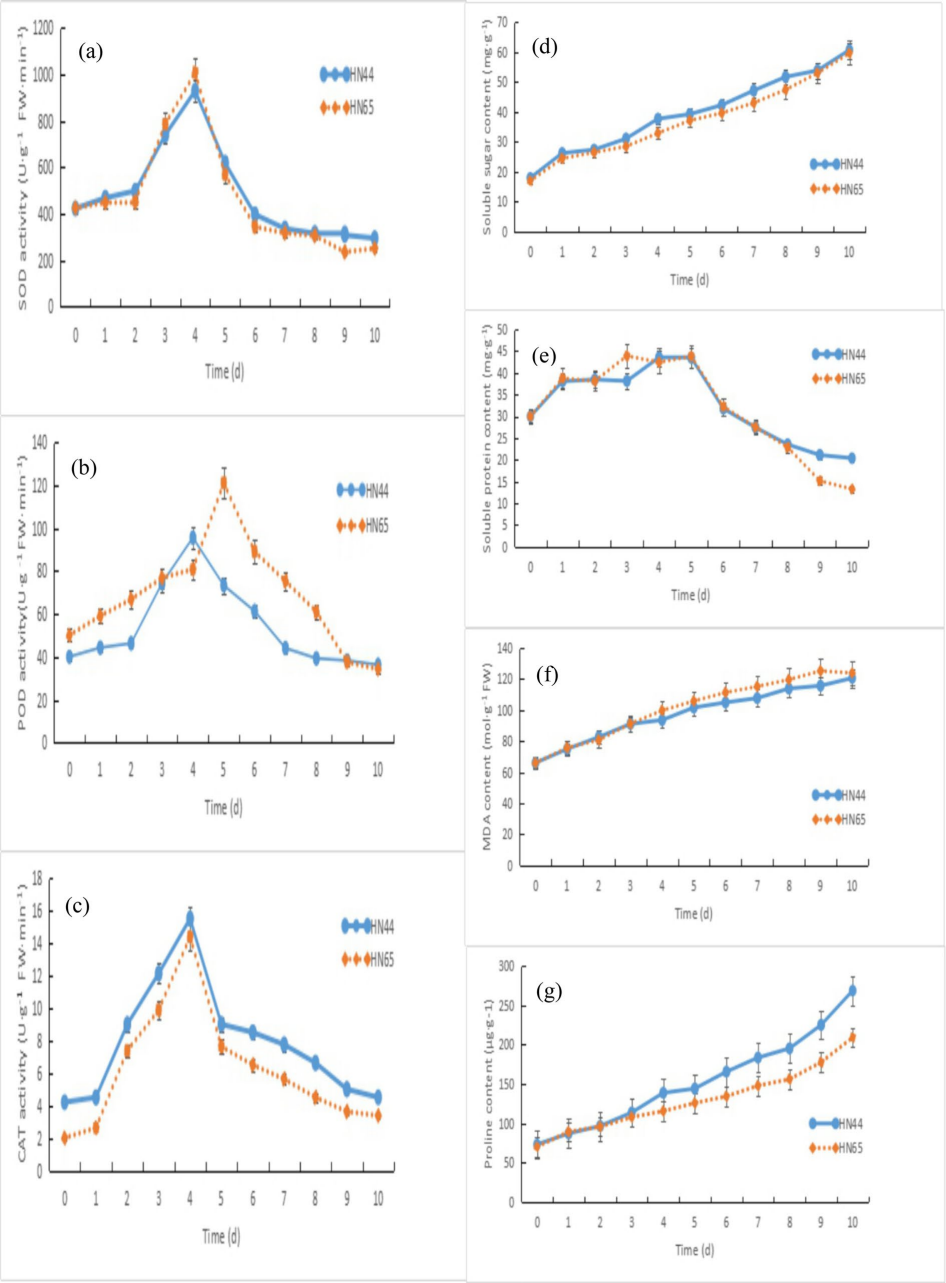
Physiological index changes in HN44 and HN65 soybean plant varieties. **a** superoxide dismutase activity; **b** peroxidase activity; **c** catalase activity; **d** soluble sugar content; **e** soluble protein content; **f** malondialdehyde content; **g** proline content

As shown in Fig. 1, on the fourth day of PEG-6000-induced drought stress, the leaves of initial pod stage soybean plant varieties HN44 (drought-resistant) and HN65 (drought-sensitive) exhibited slightly curled edges and partially yellowed surfaces with signs of water loss. Figure 2a and c display the similar SOD and CAT enzyme activity level change trends, which increased from days 0 to 4, followed by a decline. Figure 2d showed the changes of soluble sugar and proline content in the two soybean plant varieties were also similar, with content of each increasing without decline from days 0 to 10. Figure 2b compares the changes in POD activity levels: HN44 POD activity exhibited an increase from days 0 to 4, followed by a decline, while HN65 POD activity did not peak until day 5. Figure 2e compares the changes in soluble protein content: HN44 soluble protein content decreased slightly on days 3 and 5, and continued to decline after day 5, while HN65 soluble protein content dipped on days 2 and 4 and continued to decline after day 5. Figure 2f compares the changes in malondialdehyde (MDA) content: the HN44 MDA content increased continuously from days 0 to 10, while the HN65 MDA content increased from days 0 to 9, followed by a slight decrease on day 10. Figure 2g showed the change of proline content. From 0 to 10 days, the content of HN44 was always higher than that of HN65. The CAT, proline and soluble sugar contents of HN44 were always higher than those of HN65, and MDA was lower than that of HN65. Other antioxidant enzymes and osmotic adjustment substances had little difference. This indicated that HN44 was less damaged than HN65 under drought stress and had stronger drought resistance. Based on these physiological index changes, day 4 soybean leaves were selected to study the changes in metabolomics.

### Qualitative and quantitative analysis of metabolites

To ensure the repeatability of samples at different drought stress duration, quality control (QC) samples were inserted after every 10 test samples (Li and Song 2019). The overlap diagram of total ion flow (TIC) with different QC samples (Additional file 1: Fig. S1) demonstrates a high TIC overlap, with identical retention time and peak intensity, which indicates that the sample signal stability improved with separated samples.

Based on the Kyoto Encyclopedia of Genes and Genomes (KEGG) compound database, the MetWare database, and multiple reaction monitoring, the metabolites of drought stress samples were analyzed qualitatively and quantitatively by mass spectrometry. A total of 440 metabolites were detected in HN44 using a widely targeted metabolic analysis (Additional file 3: Table S1). A total of 441 metabolites were identified in HN65 (Additional file 4: Table S2).

### Principal component analysis

**Fig. 3:**
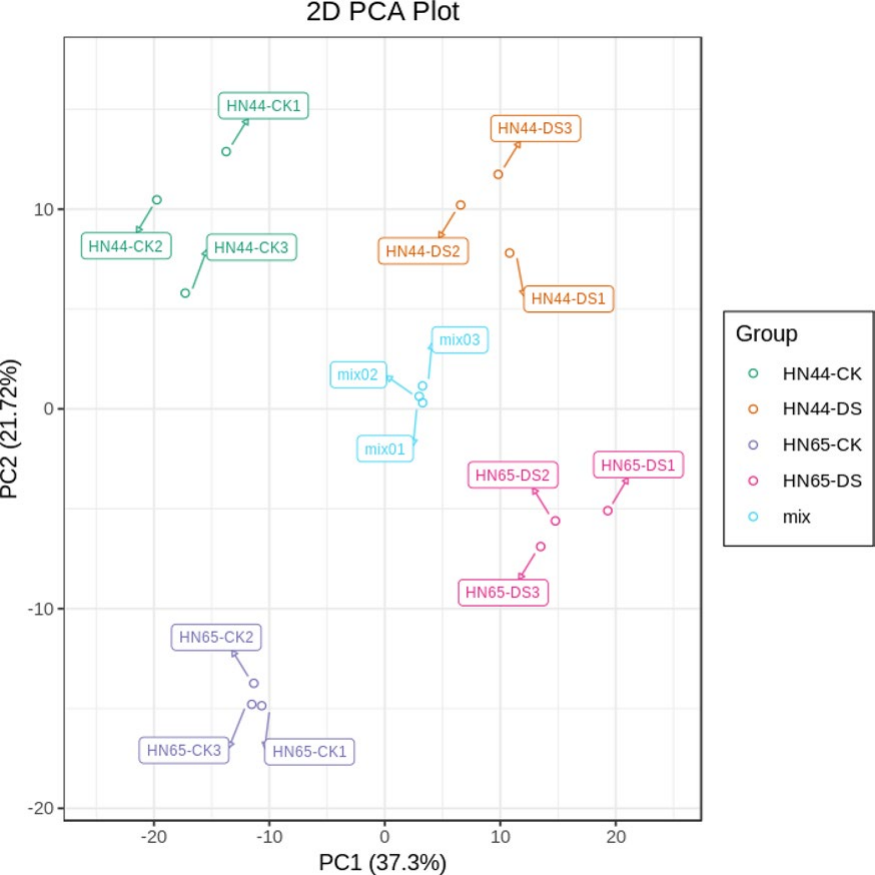
PCA score chart. The X-axis represents the first principal component and the Y-axis represents the second principal component. The closer the distance between the samples, the better the repeatability

Principal component analysis (PCA) was performed to provide a preliminary understanding of the overall intra- and intergroup metabolic differences among the HN44-CK, HN44-DS, HN65-CK, HN65-DS groups. The sample aggregation within each group indicated good repeatability, the sample distances and differences were small. There was virtually no discrepancy among the samples within each drought stress group, while an obvious divergence between DS and CK groups was revealed. The PCA score chart in Fig. 3 suggests notable metabolic differences among plant states at different points of drought stress duration. We also conducted a paired analysis of the metabolic differences between the two soybean plant varieties during drought treatment. The first component (PC1) scores for HN44 and HN65 varieties were 47.85% and 57.52% (Additional file 2: Fig. S2), respectively, with metabolism during drought treatment between the two varieties significantly separated. This indicates the significant effect of the drought stress treatment on soybean plant metabolism, with a significantly greater effect on HN65 than on HN44.

### Orthogonal partial least squares discriminant analysis

**Fig. 4:**
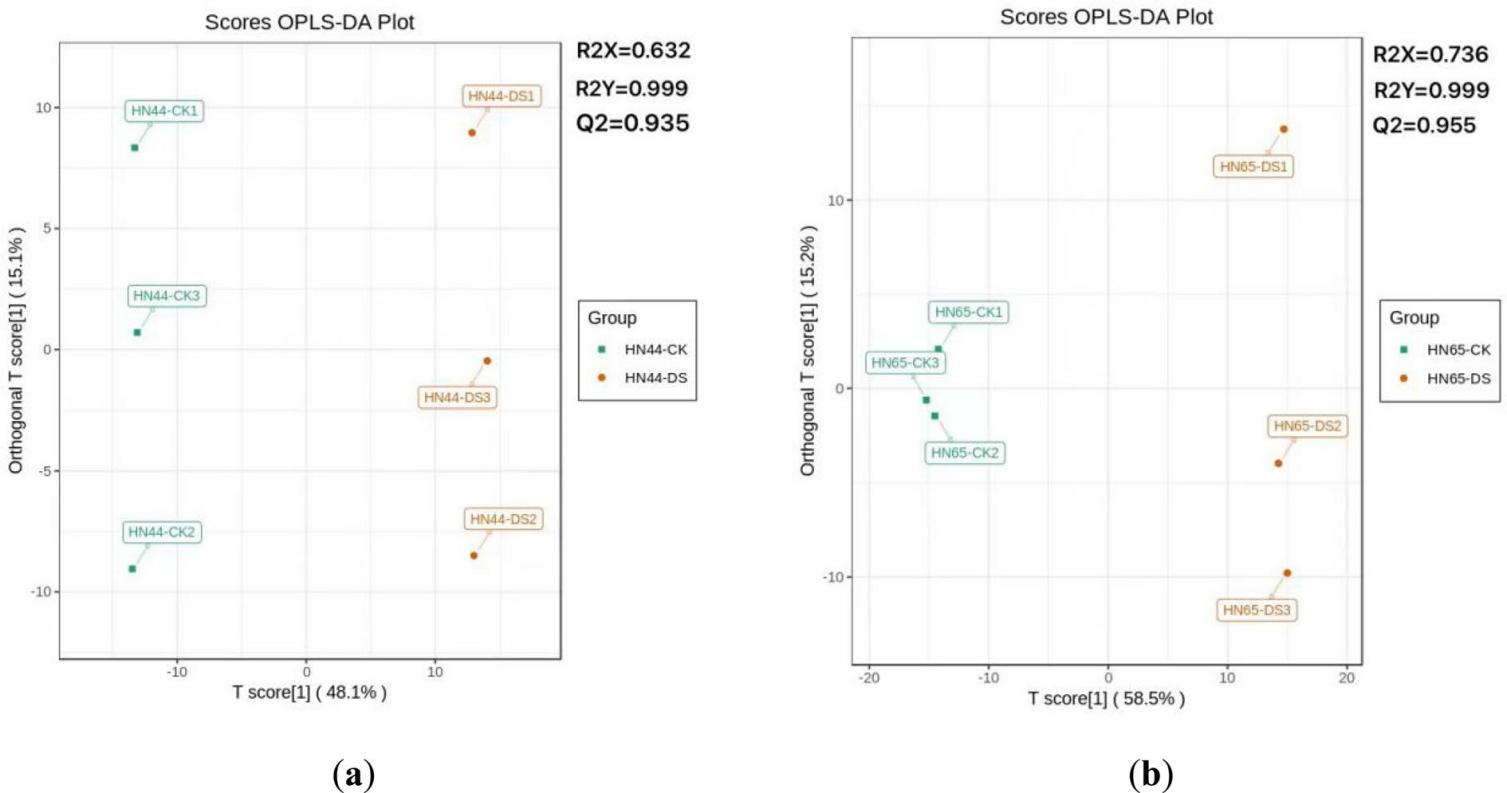
OPLS-DA score plots between the following pairs of groups: **a** HN44-CK vs. HN44-DS; **b** HN65-CK vs. HN65-DS. Note: R^2^X, R^2^Y and Q^2^ indicate original model values, the abscissa indicates the similarity between Y after replacement and original Y, and the ordinate indicates R^2^Y and Q^2^ values. The evaluation model prediction parameters were R^2^X, R^2^Y and Q^2^

The results showed that in the four groups of samples, R^2^X, R^2^Y, and Q^2^ were higher than 0.632, 0.999, and 0.935, respectively, confirming the different metabolite drought stress responses (Fig. 4). The OPLS-DA model was validated by 200 random permutation and combination comparison tests (Additional file 2: Fig. S2). Both R^2^Y' and Q^2^' are smaller than R^2^Y and Q^2^ of the original model, indicating that the model is meaningful. Subsequently, differential metabolites were screened according to the VIP value analysis.

### Screening and analysis of differential metabolites

**Fig. 5:**
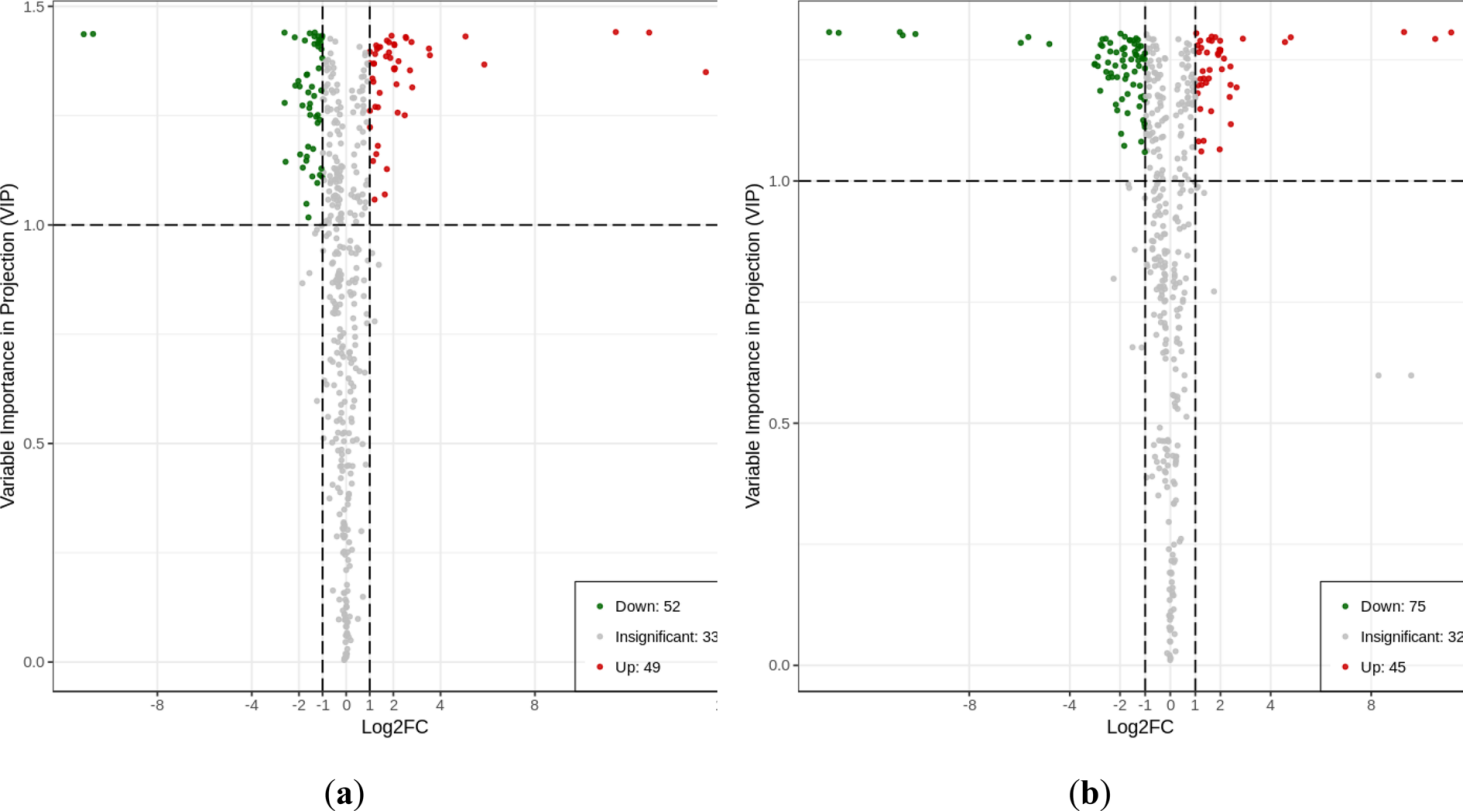
Volcano maps of differential metabolites between the following pairs of groups: **a** HN44-CK vs. HN44-DS; **b** HN65-CK vs. HN65-DS. Each volcanic map point represents a metabolite; Abscissa, compare the logarithm of the number fold change of metabolites in the sample; Ordinate, VIP value. Increased abscissa absolute values indicate increased differences in expression multiples between the two samples, and increased ordinate values indicate increased significance in differential expression; metabolites with more reliable differential expression were screened

The fold change was combined with the VIP value of OPLS-DA model to screen differential metabolites. Metabolites with change multiple ≥ 2 and ≤ 0.5 were selected, and metabolites with VIP ≥ 1 were selected as differential metabolites. The results produced 101 differential metabolites (49 upregulated and 52 downregulated) in HN44-CK vs. HN44-DS, 120 differential metabolites (45 upregulated and 75 downregulated) in HN65-CK vs. HN65-DS. Figure 5 shows a volcanic map of paired comparisons of differential metabolites.

### Change rule of metabolites

**Fig. 6:**
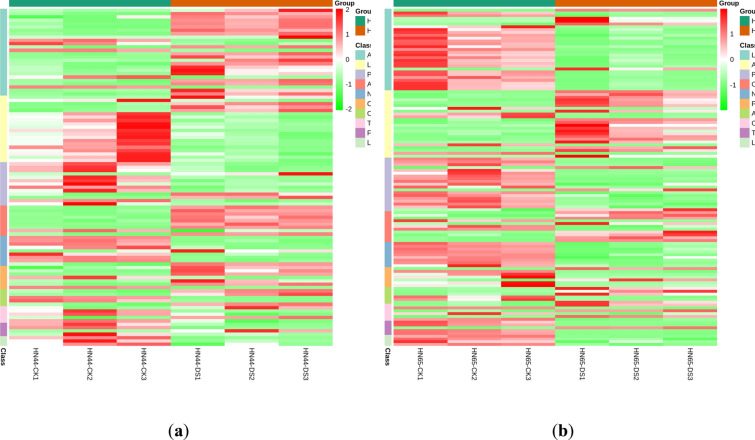
Clustering heat maps of differential metabolites between the following pairs of groups: **a** HN44-CK vs. HN44-DS; **b** HN65-CK vs. HN65-DS. The abscissa represents the sample name; the ordinates represent differential metabolites and hierarchical clustering results. Red indicates high content; Green, low content

To facilitate the observation of changes in metabolites, we used unit variance scaling (UV) normalization of metabolites with significant differences, using the pheatmap package of R software to draw the heat map. Using the cluster thermal analysis of metabolites detected in all samples shown in Fig. 6, we classified the differential metabolites into 10 categories: flavonoids, lipids, phenolic acids, amino acids and their derivatives, nucleotides and their derivatives, organic acids, alkaloids, terpenoids, lignans, coumarins and other substances (OS). The metabolite contents in six samples of the four groups slightly varied, but the differences were not significant. Drought-subjected HN44 soybean plants exhibited increased contents of amino acids and their derivatives, alkaloids, and organic acids and decreased contents of lipids, phenolic acids, nucleotides and their derivatives, lignin, and coumarins, when compared to the HN44 control group. Drought-stressed HN65 exhibited increased contents of amino acids and their derivatives and OS, with a slight increase in alkaloid content, and a decrease in the content of organic acids, lipids, phenolic acids, nucleotides and their derivatives, lignin, and coumarins when compared to the HN65 control group.

### Changes of the top 20 differential metabolites

**Fig. 7:**
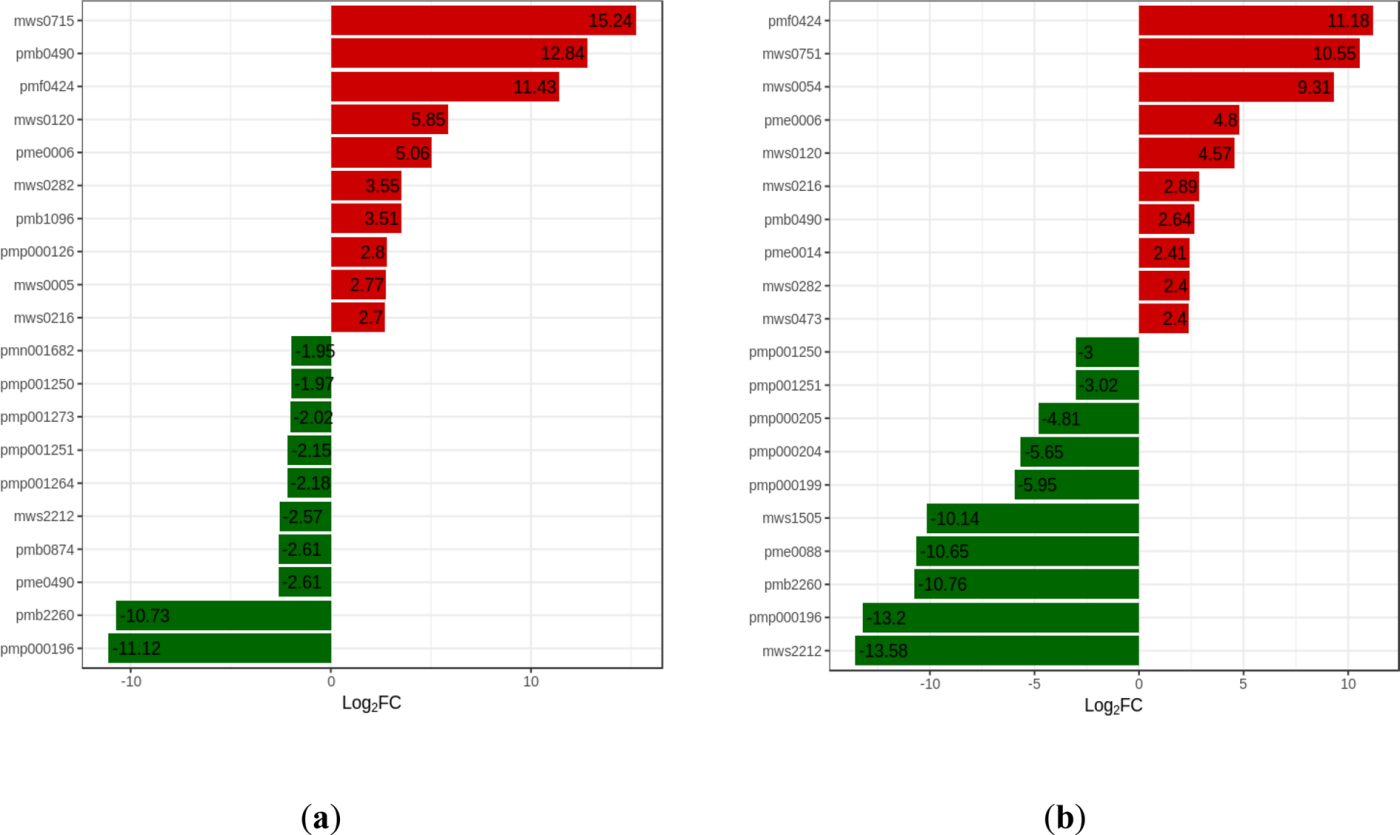
Column graph of log_2_FC values of the top 20 differential metabolites. **a** HN44-CK vs. HN44-DS; **b** HN65-CK vs. HN65-DS. The abscissa is the log2FC of the differential metabolites, and the ordinate is the differential metabolites. Red represents up-regulation, green represents down-regulation

We compared the changes in the top 20 differential metabolites between the two varieties (Fig. 7) to find the reasons for HN44’s stronger drought resistance. We found that in the up-regulated substances, six substances (Isochlorogenic acid B, l-Proline, Trans-4-Hydroxy-l-proline, N-p-Coumaroyl putrescine, l-Tryptophan, Choline alfoscerate) were accumulated between the two varieties, and the change multiples were basically high in HN44, but N-p-Coumaroyl putrescine was quite different between the two varieties. The fold change reached 7336.07 in HN44 and only 6.24 in HN65. In addition, the maximum fold change of Phenylacetyl-l-glutamine in HN44 reached 38,737.31. Among the down-regulated substances, five substances [Caffeic acid, LysoPC 15: 1, Soyasaponin γa, PC (18: 2) isomer, PC (18: 2)] were accumulated between the two varieties, and the fold change was higher in HN65. However, Caffeic acid was quite different between the two varieties. The Log_2_FC value reached − 13.58 in HN65 and only − 2.57 in HN44.

### KEGG enrichment analysis of differential metabolites

**Fig. 8:**
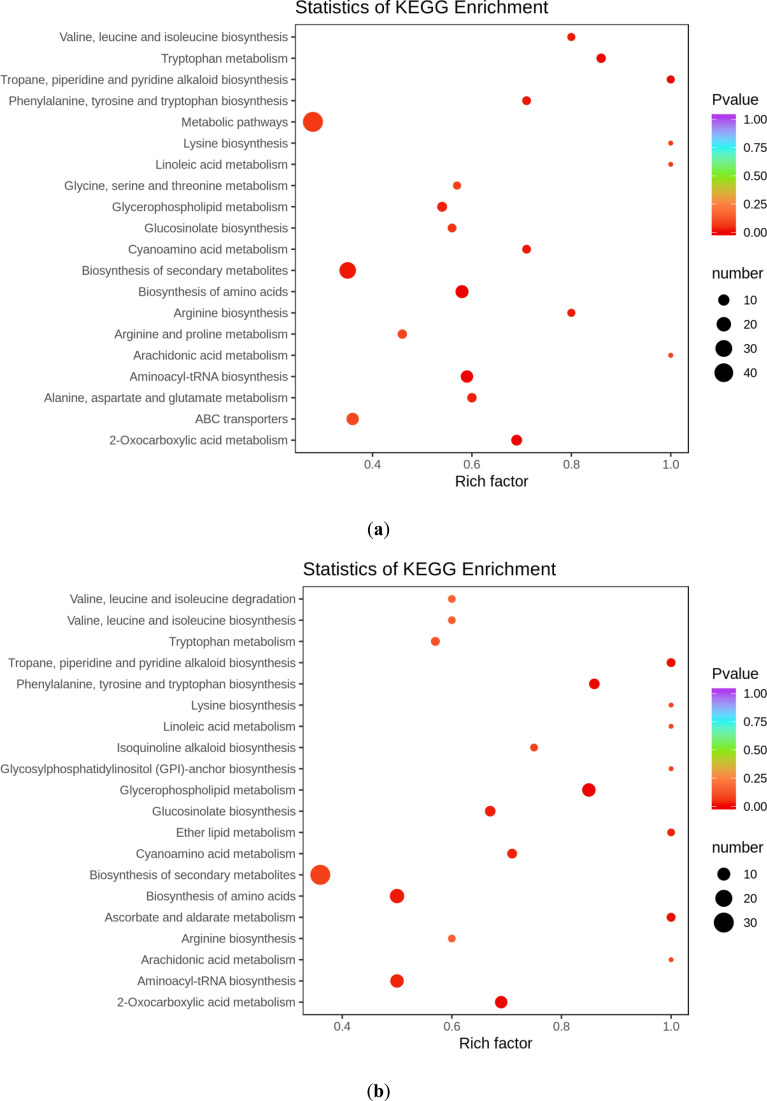
KEGG enrichment diagrams of differential metabolites. **a** HN44-CK vs. HN44-DS; **b** HN65-CK vs. HN65-DS. The abscissa represents the Rich factor corresponding to each pathway, the ordinate is the pathway name, the color of the point is the pvalue, and the redder the more significant the enrichment. The size of the dots represents the number of enriched differential metabolites

Differential metabolism interactions within organisms form different pathways, and to identify the pathways relevant to this study, we used the KEGG database to annotate and display the differential metabolites (Fig. 8). In HN44, the five pathways with the most significant enrichment were biosynthesis of amino acids, of aminoacyl-tRNA, 2-Oxocarboxylic acid metabolism, tropane metabolism, and tropane, piperidine and pyridine alkaloid biosynthesis. Similarly, in HN65, the five pathways with the most significant enrichment were glycerophospholipid metabolism, 2-Oxocarboxylic acid metabolism, phenylalanine, tyrosine and tryptophan biosynthesis, ascorbate and aldarate metabolism, tropane, piperidine and pyridine alkaloid biosynthesis. 2-Oxocarboxylic acid metabolism is closely related to TCA cycle. Tricarboxylic acid cycle is the final metabolic pathway of the three nutrients (sugars, lipids, and amino acids). Amino acid metabolism and lipid metabolism play a key role in drought resistance of the two varieties, respectively. Then the difference in drought resistance may be caused by the differences in metabolites in these different metabolic pathways. All these pathways have a positive role in addressing droughts.

### Drought-induced metabolic pathways of amino acids and their derivatives

The powerful differential expression of amino acids and their derivatives facilitate the coping mechanisms of soybean plants in response to drought stress. In the drought-resistant variety HN44, we detected the upregulation of 21 and the downregulation of five amino acids and their derivatives. In the drought-sensitive variety HN65, 19 of 24 detected amino acids and their derivatives were upregulated. The up-regulated metabolites of HN44 were more abundant. This represented the improvement of drought resistance.

**Fig. 9:**
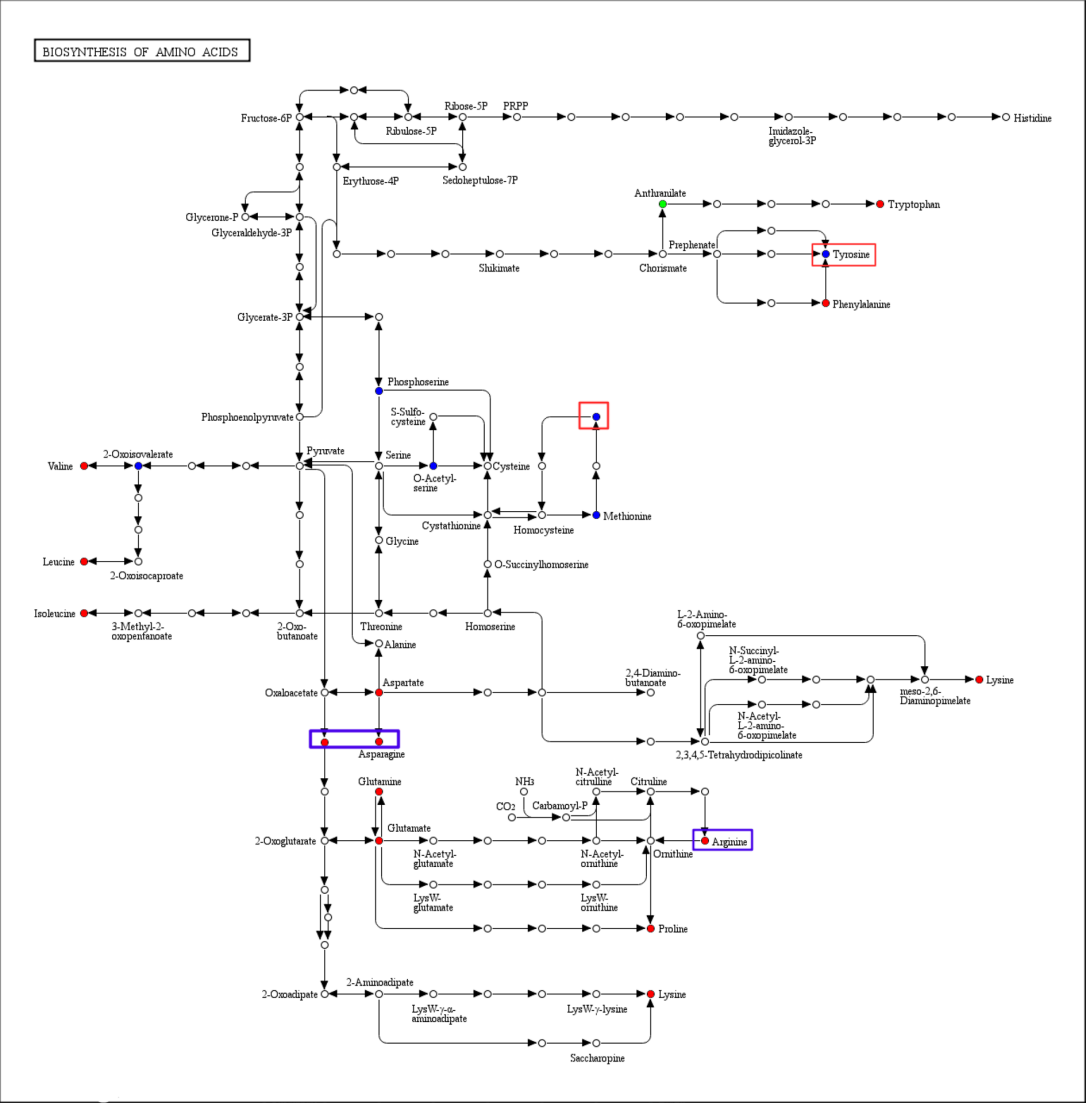
Synthesis pathway of amino acids. Red indicates a significant increase in metabolite content in the experimental group, blue represents the metabolite was detected but no significant change, and green indicates a significant decrease in metabolite content in the experimental group. The red boxes represent that the metabolite content is significantly up-regulated in HN65, and the blue boxes represent that the metabolite is detected in HN65 but have no significant change

We focused on amino acid-related metabolic pathways to find differences between the two varieties (Fig. 9). Under drought conditions, Tryptophan, l-Phenylalanine, l-Valine, l-Isoleucine, l-Leucine, l-Lysine, l-Arginine, l-Proline, l-Lysine, l-Glutamate, l-Glutamine, and l-Aspartate in the two varieties were up-regulated, and only Anthranilate was down-regulated in the two varieties. These amino acids play an important role in response to drought in two varieties. Different from HN44, l-Tyrosine and S-Adenosylhomocysteine were up-regulated in HN65, and there was no significant change in HN44. The other two substances, l-Asparagine and Citric acid, were significantly up-regulated in HN44, and there was no significant change in HN65. In particular, Citric acid was not only involved in the metabolism of multiple amino acids, but also an important substance involved in the TCA cycle. When HN44 was subjected to drought, the content of Citric acid increased. The fold change reached 2.68, which may be one of the important reasons why HN44 had stronger drought resistance than HN65.

### Difference in lipid metabolites between two varieties

Since glycerophospholipid metabolism is one of the important ways to cope with drought in HN65, we also studied the difference of lipid metabolites between the two varieties. Under drought conditions, 20 lipid substances were detected in HN44 (16 down-regulated, 4 up-regulated), 29 lipid substances were detected in HN65 (24 down-regulated, 5 up-regulated), most of these down-regulated substances belong to LPC, and up-regulated substances most were free fatty acids. It is worth noting that the up-regulated substance tridecanoic acid is quite different between the two varieties, with a fold change of 1496.1 in HN65 and 3.5 in HN44, indicating that tridecanoic acid plays an important role in the drought resistance of HN65. However, when tridecanoic acid was annotated into the KEGG database, no metabolic pathways related to it were found, so its mechanism of action is still unclear.

### Drought-induced secondary metabolite accumulation

In the HN44-DS samples, 13 phenolic acids, 4 flavonoids, 9 alkaloids, 5 terpenoids, and 7 organic acids were detected. Of these secondary metabolites, upregulation of the following was detected: 3 phenolic acids including p-coumasterol, rosmarinic acid, and isochlorogenic acid b; flavonoid luteolin-6,8-di-C-glucoside; 8 alkaloids including tryptamine, indole-5-carboxylic acid, indole-3-formaldehyde, N-acetyl-5-hydroxytryptamine, 3-indole carboxylic acid, N-p-coumaroyl putrescine, indole, and N-benzylidenemethylamine; 2 terpenoids including soybean saponin αa and soybean saponin Be; and 5 organic acids including β-hydroxyisovalerate, citric acid, 2-methylsuccinic acid, galactose aldehyde acid, and 6-aminohexanoic acid.

In the HN65-DS samples, 19 phenolic acids, 7 flavonoids, 6 alkaloids, 5 terpenes, and 6 organic acids were detected. Of these secondary metabolites, upregulation of the following was detected: 3 phenolic acids including oleic acid 11-methyl ester, p-coumaroyl, and isochlorogenic acid b; 2 flavonoids including apigenin-8-C-glucoside and catechin; 4 alkaloids including N-p-coumaroylputrescine, N-phenylmethylene iso methylamine, tryptamine, and indole; terpene soybean saponin Be; 5 organic acids including α-D-galacturonic acid, 2-methylsuccinic acid, and 6-aminocaproic acid. These secondary metabolites also played an important role in drought resistance of soybean.

## Discussion

Drought is usually caused by rainfall pattern, greenhouse effect and temperature change. It is an important stress factor limiting plant growth, regulation and distribution (Caser et al. 2018). However, plants effectively use their components and energy to combat various stressors and preliminary studies have shown that adverse conditions will cause varying degrees of physiological process regulation (Cramer et al. 2013). Studies on millet have found that oxidative damage indicators, such as proline concentration and MDA content, increase under drought stress (Mukami et al. 2019). Drought stress also increases relative conductivity, content of proline, MDA, and H_2_O_2_, and activity of reactive oxygen species-scavenging enzymes such as POD, ascorbate peroxidase (APX), and CAT, and reduced some physiological parameters, including root water content and root length, which recovered after rehydration (Cao et al. 2017). We studied the physiological changes of two soybean varieties under drought stress. With the prolongation of drought stress time, the activities of antioxidant enzymes increased first and then decreased, and the degree of membrane lipid peroxidation gradually increased. The damage of the drought-resistant variety HN44 was less than sensitive variety HN65. In plants, the accumulation and metabolism of proline are related to the abiotic stress avoidance mechanism and drought stress can stimulate free amino acids such as arginine, proline, asparagine, and amide (Furlan et al. 2020; Szabados and Savouré 2010). Under stress conditions, proline plays a protective role in supporting osmotic regulation and plant recovery from cell dehydration, and its significant accumulation under drought conditions commonly improves osmotic stress tolerance (Ozturk et al. 2021; Hayat et al. 2012). Our results showed that with the extension of drought stress duration, the accumulation of proline in plants continued to increase. In our experiment, it was found that the proline content of drought-resistant variety HN44 was always higher than that of HN65, and two prolines (Trans-4-Hydroxy-l-proline and l-Proline) were detected in HN65 at the metabolic level. Their fold change reached 7.42 and 27.79 in HN65, and their fold change reached 6.51 and 33.32 in HN44, but this only reflected the changes within the variety and could not explain the content difference between the varieties. The content of proline in plants under drought stress is high, indicating that proline plays an important role in the response to drought stress.

Amino acids and their derivatives can act as osmotic protection agents to help plants adapt to abiotic stress, including the alleviation of drought stress. In a study on tea plants, exogenous arginine and valine significantly reduced drought-induced damage in the tea cultivar Fuyun6 (Zulfiqar et al. 2019; Zhang et al. 2020b). Rabara et al. (2017) reported that 33% of the common differential metabolites in tobacco and soybean under drought stress were amino acids. Zhang et al. (2014) revealed that amino acids and derivatives involved in drought stress primarily included proline, l-isoleucine, l-asparagine, and l-valine. Guo et al. (2020) found that l-isoleucine, l-tyrosine, l-phenylalanine, l-glutamic acid, l-cysteinylglycine, and fructose-lysine accumulated in wheat under drought conditions. Carbohydrates, organic acids, and amino acids have also been reported as the main metabolic groups in drought stressed wheat (Guo et al. 2018). The accumulation of tryptophan, l-phenylalanine, l-valine, l-isoleucine, l-lysine, l-proline between the two varieties was also found in our experiments. In the present study, 26 and 24 amino acids and their derivatives were identified in HN44 and HN65, respectively, accounting for 25.7% and 20% of the total differential metabolites, respectively. The multiple reports of amino acids and their derivatives accumulating and acting in response to drought combined with our results prove that amino acids and their derivatives are the main factors in plant drought stress response.

Through the secondary metabolic pathway, plants produce a large number of plant secondary metabolites, which are a large class of compounds with diverse and highly complex structures and which are derived from primary metabolites or as intermediates metabolites in primary biosynthetic pathways (Jørgensen et al. 2005; Piasecka et al. 2015). Plant secondary metabolites are divided into several macromolecular families according to their biosynthetic pathways: phenols, terpenoids, steroids, alkaloids, and flavonoids (Kessler and Kalske 2018). Secondary metabolites also actively participate in plant drought stress response, as evidenced by the detection of phenolic acids and flavonoids in amaranth under drought stress, with significant increases in 16 phenolic acids and flavonoids proportional to drought stress severity (Sarker and Oba 2018). Salicylic acid, vanillic acid, gallic acid, chlorogenic acid, trans cinnamic acid, rutin, isoquercetin, m-coumaric acid and p-hydroxybenzoic acid are the most abundant phenolic compounds in VA3 amaranth. The accumulation of isochlorogenic acid b in both varieties was also observed in this study. Turtola et al. (2003) found that under severe drought treatment, monoterpene and resin acid levels were, respectively, 39% and 32% higher in Scotch pine seedlings and, respectively, 35% and 45% higher in Norway spruce seedlings than in control groups of each pine variety. Yahyazadeh et al. (2018) studied the change in alkaloid content in *L. chinensis* under drought conditions by comparing the berberine content in new and old leaves. Initially, young leaves accounted for approximately one-third of the total berberine content in the plant structure above ground. As the plant alkaloid content increased, the berberine level increases in new and old leaves were similar. However, at the end of the drought stress period, the percentage of berberine in young leaves had increased to over two-fifths of the total berberine content in the whole aerial plant. In the present study, a large number of secondary metabolites had accumulated under drought conditions, with 38 of 101 and 43 of 120 secondary metabolites found in HN44 in HN65, respectively. Thus, these secondary metabolites evidently also play key roles in drought stress response.

Improving plant resistance capability is an important element of cultivating improved varieties. Lipid molecules are closely related to drought resistance and cold resistance in plants (Higashi and Saito 2019). Based on the association analysis of transcriptome and lipid groups, Zhang et al. (2020a) constructed a metabolic model of the peanut regulatory cold tolerance mechanism, in which the advanced lipid system plays a central role. Under heat stress, drought conditions will lead to the accumulation of phospholipids and glycolipids (such as phosphatidylic acid, phosphatidylcholine, phosphatidylinositol, phosphatidylglycerol and digalactosylglycerol), which are involved in membrane stability and stress signal transduction (Zhang et al. 2019c). Perlikowski et al. (2020) analyzed the lipid groups of Festuca arundinacea roots under drought conditions and determined the subsequent accumulation levels of root membrane phosphoglycerin, including phosphatidylcholine (PC), phosphatidylethanolamine (PE), phosphatidylinositol (PI), and phosphatidylserine (PS). The results showed that the accumulation of most lipids, especially highly unsaturated lipids, decreased after 21 days of water deprivation. In our study, 20 and 29 lipid differential metabolites were identified in soybean varieties HN44 and HN65, respectively. These metabolites were chiefly phosphatidylcholines, lysophosphatidylcholines, lysophosphatidylethanolamines, free fatty acids, sphingolipids, and glycerol esters. Although these lipids also accumulate during and play an important role in drought resistance, the mechanism is not clear, and requires future research.

Understanding the differences in drought tolerance among plants can provide useful clues for genetic improvement of plant drought tolerance. A study of sesame seeds found that drought-tolerant varieties contained higher levels of ABA, proline, and arginine (You et al. 2019). Physiological indicators and metabolites produced the same results in our study. There are higher proline content and more abundant amino acids in HN44. In this study, metabolomics and physiology were used to find the differences in drought tolerance among plants. It was found that the CAT and soluble sugar contents of drought-tolerant variety HN44 were higher than those of sensitive variety HN65, and the MDA content was lower than that of HN65. Combined with the results of metabolomics, we believe that amino acid metabolism under drought conditions is a more powerful drought resistance pathway than lipid metabolism, but TCA cycle is still the core metabolic pathway for drought resistance in different varieties.

## Conclusions

By comparative metabolomic analyses of two soybean varieties with different drought tolerance levels, we identified a total of 101 of 440 and 120 of 441 drought stress resistance metabolites in drought-resistant HN44 and drought-sensitive HN65 soybean plant varieties, respectively. KEGG enrichment analysis indicated that these differential metabolites are involved in the biosynthesis of amino acid, glycerophospholipid metabolism and the metabolism of 2-oxocarboxylic acid. Additionally, We think that the reasons of drought resistance among soybean varieties are as follows: (1) the main metabolic pathways are different under drought stress; (2) the contents of metabolites in these metabolic pathways are different; and (3) some physiological indexes are different, such as MDA, CAT, proline content and so on.

## Supplementary Information

**Additional file 1: Figure S1.** QC sample mass spectrometry detection TIC overlay. **a** HN44-CK vs. HN44-DS; **b** HN65-CK vs. HN65-DS.

**Additional file 2: Figure S2.** OPLS-DA Verification Diagram. **a** HN44-CK vs. HN44-DS; **b** HN65-CK vs. HN65-DS. Note: The annotations in the upper left corner of the figure are R2X, R2Y and Q2 of the original model, and the abscissa is the similarity between the model Y after replacement and the original Y. The vertical axis is the R2Y and Q2 values.

**Additional file 3: Table S1.** Metabolites identified in HN44.

**Additional file 4: Table S2.** Metabolites identified in HN65.

## Data Availability

All data generated or analysed during this study are included in this published article [and its Additional files].
