# Supplementary figures and images for: Physiology and metabonomics reveal differences in drought resistance among soybean varieties
Source: Bot Stud. 2022 Mar 25;63:8. doi: 10.1186/s40529-022-00339-8 (PMC8948310; doi:10.1186/s40529-022-00339-8)

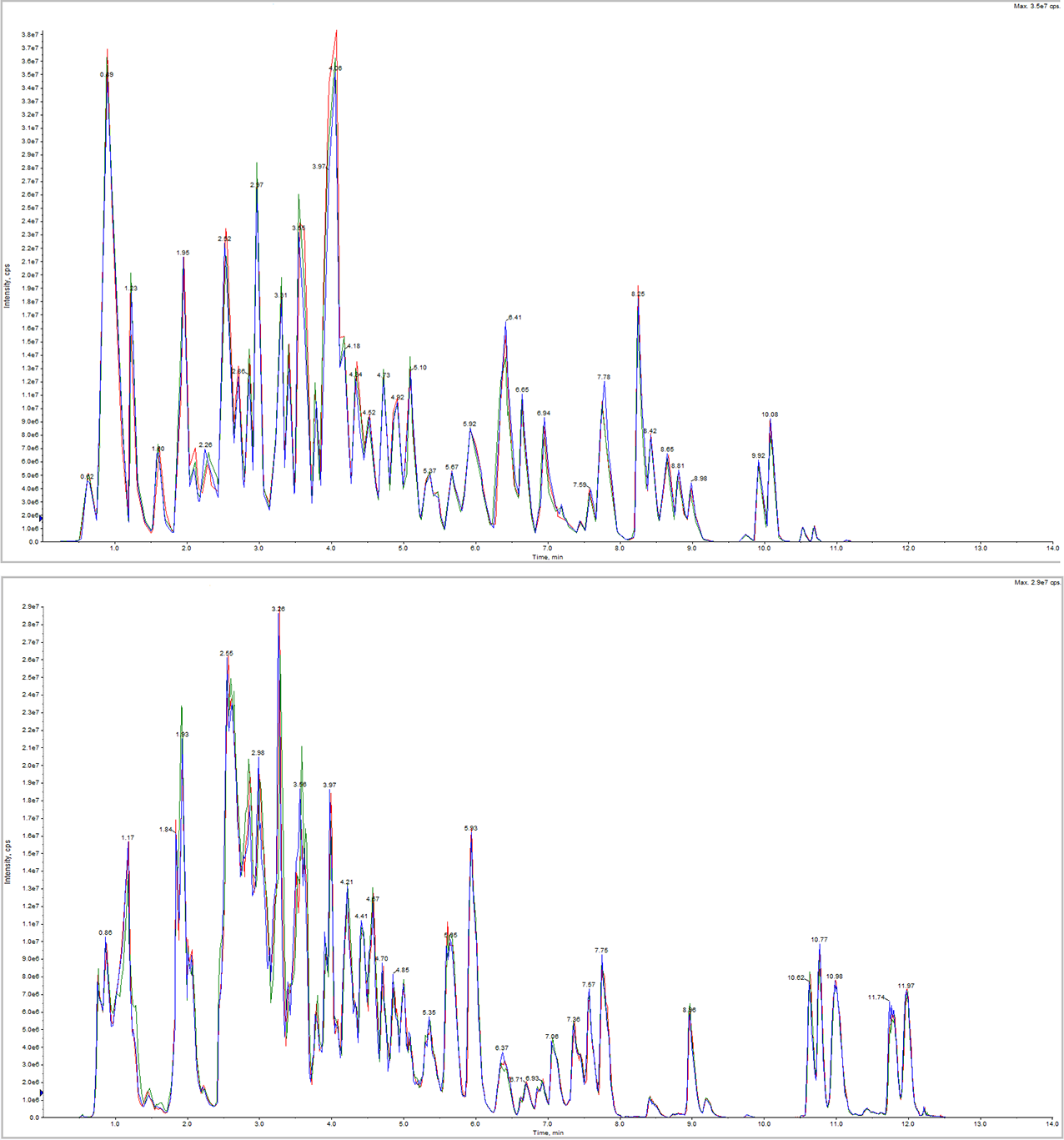

Supplement: Supplementary file 1 — Additional file 1: Figure S1. QC sample mass spectrometry detection TIC overlay. a HN44-CK vs. HN44-DS; b HN65-CK vs. HN65-DS. [file 40529_2022_339_MOESM1_ESM.png]

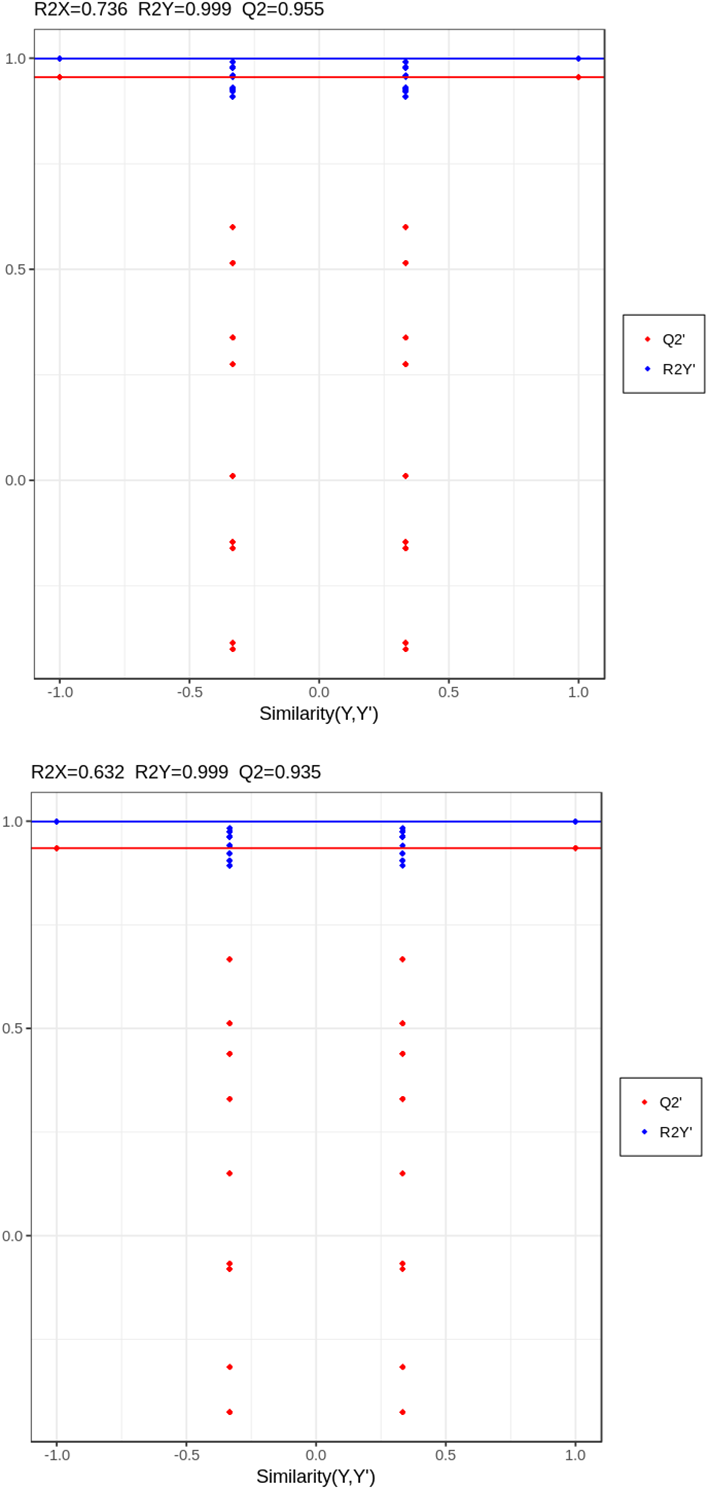

Supplement: Supplementary file 2 — Additional file 2: Figure S2. OPLS-DA Verification Diagram. a HN44-CK vs. HN44-DS; b HN65-CK vs. HN65-DS. Note: The annotations in the upper left corner of the figure are R2X, R2Y and Q2 of the original model, and the abscissa is the similarity between the model Y after replacement and the original Y. The vertical axis is the R2Y and Q2 values. [file 40529_2022_339_MOESM2_ESM.png]
